# Clinical significance of circulating microRNAs as markers in detecting and predicting congenital heart defects in children

**DOI:** 10.1186/s12967-018-1411-0

**Published:** 2018-02-27

**Authors:** Yong Song, Hilda Higgins, Jing Guo, Katrina Harrison, En Nee Schultz, Belinda J. Hales, Eric K. Moses, Jack Goldblatt, Nicholas Pachter, Guicheng Zhang

**Affiliations:** 1 School of Public Health, Curtin University of Technology, Kent St, Bentley, WA 6102 Australia; 2 Centre for Genetic Origins of Health and Disease, The University of Western Australia and Curtin University, 35 Stirling Highway, Crawley, WA 6009 Australia; 3 Genetic Services & Familial Cancer Program of WA, King Edward Memorial Hospital for Women, Subiaco, WA 6008 Australia; 4 School of Paediatrics and Child Health, The University of Western Australia, 35 Stirling Highway, Crawley, WA 6009 Australia; 5 Telethon Kids Institute, The University of Western Australia, 35 Stirling Highway, Crawley, WA 6009 Australia; 6 Curtin Health Innovation Research Institute, Curtin University, Kent St, Bentley, WA 6102 Australia

**Keywords:** Diagnostic biomarkers, MicroRNA, Congenital heart defects

## Abstract

**Background:**

Circulating microRNAs (miRNAs) are emerging as novel biomarkers for detecting cardiovascular diseases. In this study, we aimed to investigate the usefulness of miRNAs as biomarkers in diagnosing and predicting children with congenital heart defects (CHD), particularly in the context of multiple subtypes of CHD.

**Methods:**

We recruited 26 families, each having a child with CHD and parents who do not have any cardiovascular disorder. 27 families unaffected by cardiovascular disease were also included as controls. Firstly, we screened 84 circulating miRNAs relating to cardiovascular development in 6 children with atrial septal defects (ASD) and 5 healthy children. We validated the selected miRNAs with differential expression in a larger sample size (n = 27 for controls, n = 26 for cases), and evaluated their signal in different types of septal defects. Finally, we examined the identified miRNAs signatures in the parent population and assessed their diagnostic values for predicting CHD.

**Results:**

The three miRNAs hsa-let-7a, hsa-let-7b and hsa-miR-486 were significantly upregulated in children with ASD. A further validation study showed that overexpression of hsa-let-7a and hsa-let-7b was specifically present in ASD children, but not in children with other subtypes of septal defects. A similar expression profile of hsa-let-7a and hsa-let-7b was discovered in mothers of ASD children. Receiver-operating characteristic curve analyses indicated that hsa-let-7a and hsa-let-7b had significant diagnostic values for detecting ASD and in maternal samples predicting the occurrence of ASD in offspring.

**Conclusions:**

Circulating miRNAs are important markers not only for diagnosing CHD, but also for predicting CHD risk in offspring. The distinct miRNA signatures are likely to present in various subtypes of CHD, and the phenotypic heterogeneity of CHD should be considered to develop such miRNA-based assays.

## Background

With an incidence of approximately 1% in neonates, congenital heart defects (CHD) are the most common human congenital anomaly [1–3]. Such defects account for over 40% of prenatal deaths and over 20% of mortality in the first few months after birth [4]. CHD comprise a spectrum of pathology including septal defects, valve defects and lesions affecting the outflow tract. To date, approximately 20% of CHD cases are attributed to known causes such as genetic syndromes and teratogens, but very little is known about the aetiology of the remainder of cases (about 80%) [5]. This unknown aetiology of CHD follows a multifactorial inheritance model, implicating both genetic and environmental factors in disease development [5].

An accurate and early diagnosis of CHD is of great significance for timely surgical intervention and effective postnatal management. With the advent of pediatric echocardiography, accurate detection of children with CHD has significantly improved. However, it remains a challenge to use as a screening tool, particularly during routine prenatal care, with detection rates ranging from 6 to 53% due to lack of standardization [6, 7]. Therefore, development of a rapid and accurate diagnostic assay is imperative for clinical management and screening of CHD.

MicroRNAs (miRNAs) are small non-coding RNA strands that regulate the expression of genes at the post-transcriptional and translational levels. These small non-coding RNA molecules have emerged as key regulators in embryonic heart development, morphogenesis of the heart, and myocardial cell growth and differentiation [8]. In 2008, miRNAs were discovered to be present in plasma and serum with surprising stability [9]. Subsequently, several groups have reported on the use of miRNAs as circulating biomarkers for diagnosis or prognosis of cardiovascular diseases [10, 11], cancers [12, 13] and diabetes [14, 15], demonstrating that circulating miRNAs are quite specific for disease pathologies. Particularly in relation to congenital anomalies, Zhu et al. identified 4 miRNAs that were significantly up-regulated in mothers carrying foetuses with CHD [16]. In children with congenital heart malformations with ventricular septal, 8 miRNAs were dysregulated [17], while in adults with complete transposition of the great arteries after atrial switch operation, 11 miRNA expression signatures in serum were discovered, with miR-18a and miR-486-5p being associated with systemic ventricular contractility [18].

These first studies show the importance of specific miRNAs in CHD pathogenesis, and highlight the potential of circulating miRNAs as non-invasive biomarkers for detecting CHD. Nevertheless, different types of CHD possess different molecular alterations and possibly different miRNA signatures. Thus we hypothesised that specific miRNAs were associated with each subtype of congenital anomaly and that the phenotypic heterogeneity should be considered when identifying novel miRNA biomarkers and designing a sensitive diagnostic test. For the first part of this study we specifically selected children with septal defects (one of main types of CHD), but without genetic syndromes to evaluate the diagnostic values of the circulating miRNAs in the subtypes of septal defects (atrial septal defects, ASD; ventricular septal defects, VSD; atrioventricular septal defects, AVSD) in which there are pathologic and morphologic similarities. It is reported that non-syndromic CHD patients often inherit abnormal epigenetic modifications from their seemingly healthy parents [19]. On the other hand, miRNAs are believed to regulate the epigenetic machinery [20], and the regulatory effect of miRNAs is a heritable genetic trait in humans [21]. We further hypothesised that the selected miRNAs could be used as biomarkers to predict occurrence of CHD in offspring. The subsequent aim of this study was to investigate the differential expression of the specific miRNAs in unaffected parents of children with/without CHD, and assess whether the miRNA levels could discriminate parents of CHD cases from parents of normal subjects.

## Methods

### Participant recruitment

From October 2014 to July 2016, we recruited 26 children with CHD as confirmed by ultrasound technique and 27 age and gender matched healthy children as controls. The 26 CHD cases consisted of ASD (n = 12), VSD (n = 8) and AVSD (n = 6). We collected blood samples from the parents of these participants, 44 for the case group (25 mothers and 19 fathers) and 39 for the control group (23 mothers and 16 fathers). All parents in the study were healthy subjects without any cardiovascular disease.

The study was approved by the Human Research Ethics Committee at Princess Margaret Hospital for Children, Western Australia (HREC Approval No: 2014095EP). Participants were recruited from Princess Margaret Hospital in Perth, Western Australia as part of the Kids Heart Research DNA bank program; a biobank for children affected with CHD. Children with molecularly proven or clinically recognizable chromosomal or genetic syndromes causing CHD or other heart defects were excluded, as well as non-English speaking parents and pregnant mothers. A genetic counselor informed the families of the study parameters, gave them a study information package and obtained informed consent from the families that agreed to participate in the study.

### Blood sample collection and plasma preparation

Three to 5 ml peripheral whole blood samples were collected from each participant and centrifuged at 1200*g* for 10 min at 4 °C to remove the blood cells. The supernatant was transferred into microcentrifuge tubes, followed by a second centrifugation at 12,000*g* for 10 min at 4 °C to completely remove cellular components. Plasma was then aliquoted and stored at − 80 °C until miRNA isolation.

### Circulating miRNA extraction and cDNA synthesis

The total RNA in 50 μl plasma was mixed with QIAzol Lysis Reagent at a 1:5 ratio with *C. elegans* miR-39 miRNA mimic as a Spike-In Control (Qiagen Pty, Doncaster, VIC, Australia). The RNA was then extracted with the miRNeasy Serum/Plasma Kit (Qiagen), according to the manufacturer’s protocols. Purified RNA was converted into cDNA using miScript II RT Kit (Qiagen).

### miRNA PCR array assay

Six children with ASD and five controls were selected for the profiling of 84 circulating miRNAs. The miScript miRNA PCR Array for Human Cardiovascular Disease (Qiagen) was used according to the manufacturer’s instructions. This miRNA Array profiles the expression of 84 miRNAs known to exhibit altered expression during cardiovascular disease and development. PCR reactions were performed using the ViiA 7 Real-Time PCR System (Thermo Fisher Scientific, Scoresby, VIC, Australia). The reaction mixtures were incubated at 95 °C for 15 min to activate the HotStart DNA Taq polymerase, followed by 40 cycles of 94 °C for 15 s, 55 °C for 30 s and 70 °C for 30 s. The relative expression of each miRNA was normalised by the internal spike-in control Ce-miR-39. The microarray data were analysed using the miScript miRNA PCR Array Data Analysis Web Portal (http://pcrdataanalysis.sabiosciences.com/mirna/arrayanalysis.php) (Qiagen).

### Quantitative real-time PCR (qPCR)

Three miRNAs (hsa-let-7a, hsa-let-7b and hsa-miR-486) were selected from the miRNA array screening based on the significant difference (*p* value) as well as fold change between case and control. These miRNAs were further examined using qPCR in all 53 children (including the children participating in the miRNA PCR array study) and their parents (n = 83). miScript mature miRNA primer assays (Cat^#^ MS00031220, MS00003122 and MS00004284, Qiagen) were used for targeting the specific sequences of hsa-let-7a, hsa-let-7b and hsa-miR-486 respectively. The PCRs were run using a miScript Syber green PCR master mix (Qiagen) and carried out as described above with the following annealing temperatures: 55 °C for hsa-let-7a, 57 °C for hsa-let-7b, 60 °C for hsa-miR-486 and housekeeping miRNA Cel-miR-39. The specificity of amplification was verified using melting curve analysis. The expression levels of miRNAs were normalized against Ce-miR-39 using the 2^−ΔΔCT^ method, and were presented relative to values in the children or parent control group.

### Statistical analysis

Sigmaplot (version 11.0, Systat Software Inc, San Jose, USA) was used for statistical analysis. The independent T test was used to compare differences between two groups (control and CHD), whilst differences among multiple groups (control, ASD, VSD, AVSD) were assessed using one-way ANOVA, with a Tukey honestly significant difference test implemented as post hoc analysis. Correlations of miRNA expression between children and their parents were analysed using the Pearson correlation method. Data are presented as mean (SD) or median (range). In addition, receiver-operating characteristic (ROC) curves were generated for the selected miRNAs to determine their clinical utility as diagnostic biomarkers using the statistical package SPSS (version 20.0: SPSS Inc., Chicago, IL, USA). Specifically, the area under the ROC curve (AUC) was measured by computing sensitivity and specificity for each possible cut-off point of the selected miRNA expression levels. The cut-off value was determined for each individual marker to maximize the classification accuracy according to the Youden index [22]. Statistical significance was accepted as *p* < 0.05.

## Results

### Participant’s characteristics

The participants’ demographic data are listed in Table 1. There was no significant difference in general characteristics between the two groups (cases and controls) of children including age and gender. The parental age was also the similar between the case and control groups.

**Table 1:** Demographic data for the participants in the study

	Control group	Disease group	*p* value
Children’s population			
Male:female	12:18	18:8	0.29
Age (years)	7.87 ± 5.27	5.15 ± 0.50	0.58
Parent population			
Maternal:paternal	23:14	25:20	0.546
Maternal age (years)	39.15 ± 7.38	36.72 ± 6.99	0.23
Paternal age (years)	39.46 ± 7.15	37.84 ± 5.88	0.39

Data are presented as n or mean (SD)

### Selection of candidate miRNA biomarkers

**Fig. 1:**
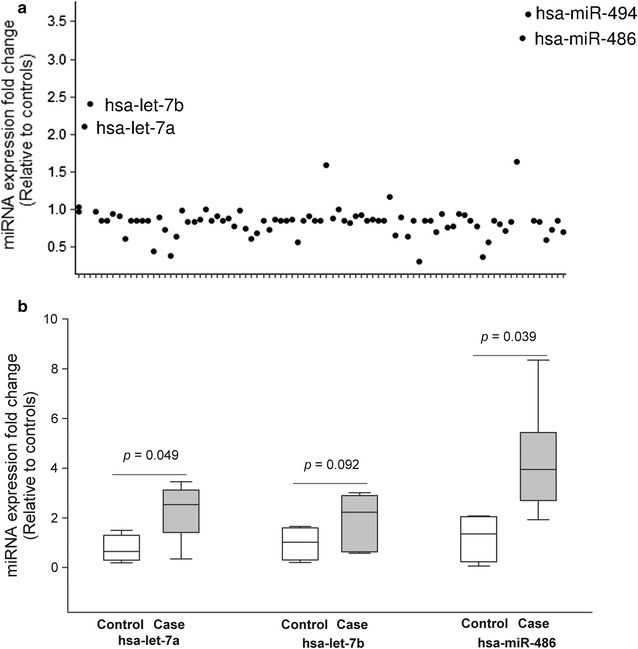
miRNA screening and selection: Eight four miRNA expression fold changes in six children with ASD (relative to five healthy children) were shown in scatter plot (**a**). The three miRNAs with high up-regulation were selected and shown in box plot (**b**). Box and whisker plots represent median with 10th and 90th centiles. *miRNA* microRNA, *ASD* atrial septal defects

Out of the 84 miRNAs that were examined (Fig. 1a), the expressions of hsa-let-7a and hsa-miR-486 were significantly up-regulated in children with ASD (*p* < 0.05) compared to the controls. We also observed a marginal increase of the hsa-let-7b level (*p* = 0.092), which was increased more than twofold for the disease group (Fig. 1b). Although hsa-miR-494 expression was up-regulated in case children by approximately 3.6-fold (95% CI 0.00001, 13.69) relative to the controls, such difference was not significant (*p* = 0.389) since hsa-miR-494 change was highly variable in the case group. No significant differences in the other plasma miRNA levels were identified between the cases and healthy controls (*p* > 0.05). The three miRNAs (hsa-let-7a, hsa-let-7b and hsa-miR-486) were selected for further analysis.

### Validation of the miRNA signatures in the children population

**Fig. 2:**
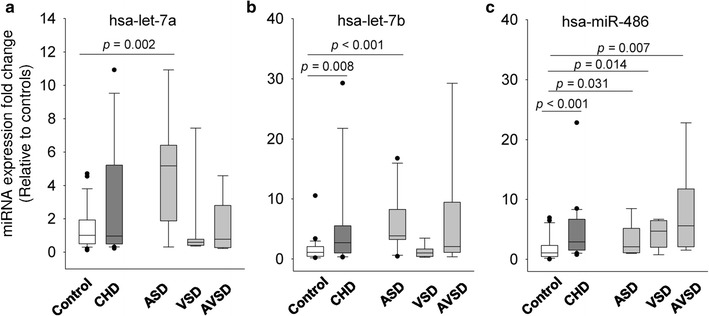
Validation of the miRNA signatures in children population: comparison of hsa-let-7a (**a**), hsa-let-7b (**b**) and hsa-miR-486 (**c**) expression between children with CHD (n = 26) and healthy controls (n = 27). CHD phenotype is further stratified into ASD (n = 12), VSD (n = 8) and AVSD (n = 6) in comparison with the controls. Box and whisker plots represent median with 10th and 90th centiles. *miRNA* microRNA, *CHD* congenital heart defects, *ASD* atrial septal defects, *VSD* ventricular septal defects, *AVSD* atrioventricular septal defects

We examined the biomarker potential of the selected three miRNAs using the full cohort of children (26 cases, 27 controls). Compared to the children of the control group, children with CHD (including the three types of septal defects) demonstrated a significantly higher level of hsa-let-7b and hsa-miR-486, whilst hsa-let-7a level did not show a significant difference between the two groups (Fig. 2). After stratifying by disease phenotype and compared to the children from the control group, children with ASD were found to have a higher expression of hsa-let-7a (*p* = 0.002) and hsa-let-7b (*p* < 0.001) (Fig. 2a, b) while the hsa-miR-486 level was significantly higher for all ASD, VSD and AVSD groups (Fig. 2c).

**Fig. 3:**
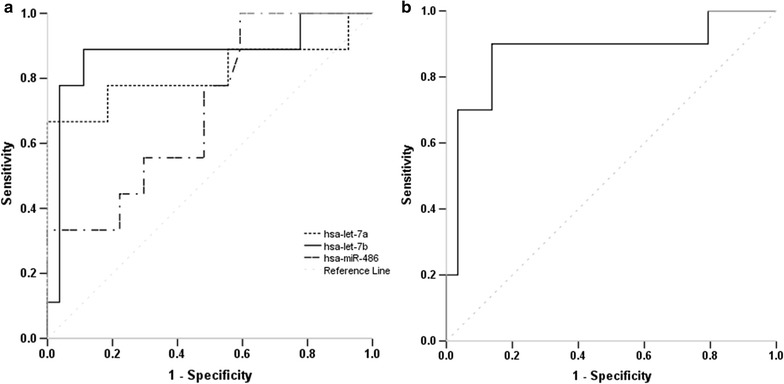
Analysis of the discriminatory power of the miRNAs in children population: the graph shows the ROC curve analysis of the validation study for hsa-let-7a, hsa-let-7b and hsa-miR-486 (**a**) and the combination of the three miRNAs (**b**) in discriminating patients with ASD from healthy controls. *miRNA* microRNA, *ROC* receiver-operating characteristic, *AUC* area under the ROC curve, *ASD* atrial septal defects

We further evaluated hsa-let-7a, hsa-let-7b and hsa-miR-486 for their ability to differentiate between healthy children and CHD children or specifically children with ASD using ROC curve analysis. As shown in Table 2, irrespective of CHD phenotype, only hsa-miR-486 showed a significant diagnostic ability with AUC value of 0.755 (*p* = 0.002), sensitivity of 69% and specificity of 70% (cut-off value: 1.39). When combining all three miRNAs, the AUC was slightly increased to a value of 0.783 (*p* = 0.001). When applying the ROC analyses to detect children with ASD (Fig. 3), a significant improvement of diagnostic accuracy was revealed for hsa-let-7a and hsa-let-7b, with AUC values of 0.833 (95% CI 0.641–1.000, *p* = 0.002) and 0.900 (95% CI 0.764–1.000, *p* < 0.001). The optimal diagnostic sensitivity and specificity were 70 and 100% for hsa-let-7a with a cut-off expression value 4.94, and 91 and 90% for hsa-let-7a with a cut-off expression value 2.30 respectively. Combining the different miRNAs did not further improve the diagnostic accuracy (Table 2).

**Table 2:** ROC curve analysis showing the discriminative power of the selected microRNAs (miRNA) or the combined form in children and maternal populations

Population	Phenotype	miRNA	AUC	95% CI	*p* value
Children	CHD	hsa-let-7a	0.544	0.368–0.720	0.601
		hsa-let-7b	0.656	0.486–0.816	0.063
		hsa-miR-486	0.755	0.622–0.888	0.002
		All three combined	0.783	0.653–0.912	0.001
Children	ASD	hsa-let-7a	0.833	0.641–1.000	0.002
		hsa-let-7b	0.900	0.764–1.000	< 0.001
		hsa-miR-486	0.806	0.516–0.895	0.068
		All three combined	0.877	0.700–1.000	0.001
Mother	ASD	hsa-let-7a	0.917	0.822–1.000	< 0.001
		hsa-let-7b	0.680	0.482–0.877	0.097
		hsa-miR-486	0.562	0.297–0.827	0.567
		hsa-let-7a/hsa-let-7b	0.909	0.810–1.000	0.050

*ROC* receiver-operating characteristic, *AUC* area under an ROC curve, *CHD* congenital heart defects, including the three types of septal defects, *ASD* atrial septal defects, *CI* confidence interval

### Further investigation of the miRNA signatures in the parent population

**Fig. 4:**
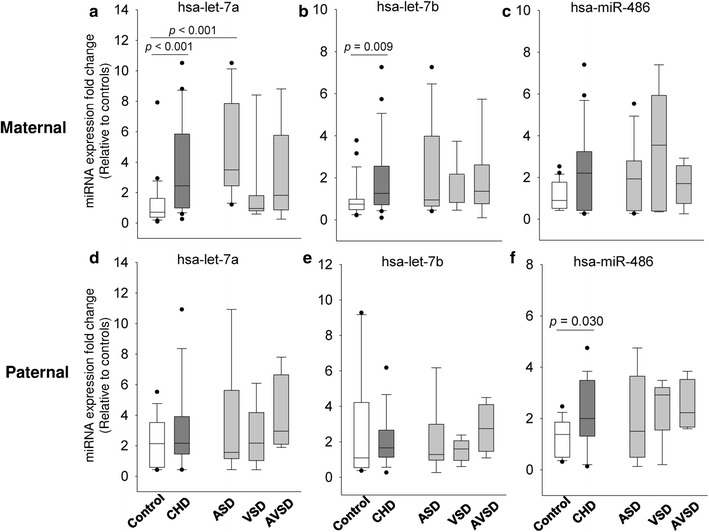
Differential expression of the miRNA signatures in parent population: Comparison of hsa-let-7a (**a**), hsa-let-7b (**b**) and hsa-miR-486 (**c**) expression between parent with and without CHD children, stratified by gender: maternal (**a**–**c**) and paternal (**d**–**f**) populations. CHD phenotype is further stratified into ASD (n = 12 for maternal group; n = 9 for paternal group), VSD (n = 7 for maternal group; n = 6 for paternal group) and AVSD (n = 6 for maternal; n = 4 for paternal group) in comparison with the control parents (n = 23 for maternal group; n = 16 for paternal group). Box and whisker plots represent median with 10th and 90th centiles. *miRNA* microRNA, *CHD* congenital heart defects, *ASD* atrial septal defects, *VSD* ventricular septal defects, *AVSD* atrioventricular septal defects

Similarly, parents of CHD children had a higher level of circulating hsa-let-7a, hsa-let-7b and hsa-miR-486 compared to parents of healthy children (*p* < 0.05, data not shown). After stratifying by gender (Fig. 4), the differences remained significant for hsa-let-7a (*p* < 0.001) and hsa-let-7b ((*p* = 0.009) in the maternal population, and for hsa-miR-486 in the paternal population (*p* = 0.03). Further stratification by disease subtype showed that mothers of ASD children had a 3.8-fold increase in hsa-let-7a, compared to mothers with healthy children (*p* < 0.001, Fig. 4a). No other significant difference was observed between the subtype groups and controls for the three miRNA expressions.

**Fig. 5:**
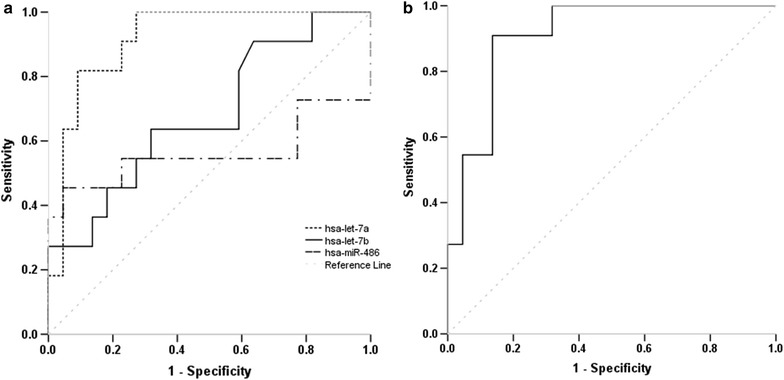
Analysis of the discriminatory power of the miRNAs in maternal population: the graph shows the ROC curve analysis of the three selected miRNAs (hsa-let-7a, hsa-let-7b and hsa-miR-486) (**a**) and the combination of hsa-let-7a and hsa-let-7b (**b**) in discriminating mothers with ASD children from mothers with healthy children. *miRNA* microRNA, *ROC* receiver-operating characteristic, *AUC* area under the ROC curve, *ASD* atrial septal defects

ROC curve analysis (Fig. 5a) showed that hsa-let-7a had the best diagnostic performance with an AUC of 0.917 (95% CI 0.822–1.000, *p* < 0.001; Table 2). At a cut-off of 2.42, the hsa-let-7a level could distinguish between mothers with ASD offspring and mothers of healthy children, with a sensitivity of 82% and a specificity of 91%. The other two miRNAs or combined miRNAs did not demonstrate a significant or better discriminative value (Fig. 5b and Table 2).

### Correlation of the miRNA expression in children and parent populations

**Fig. 6:**
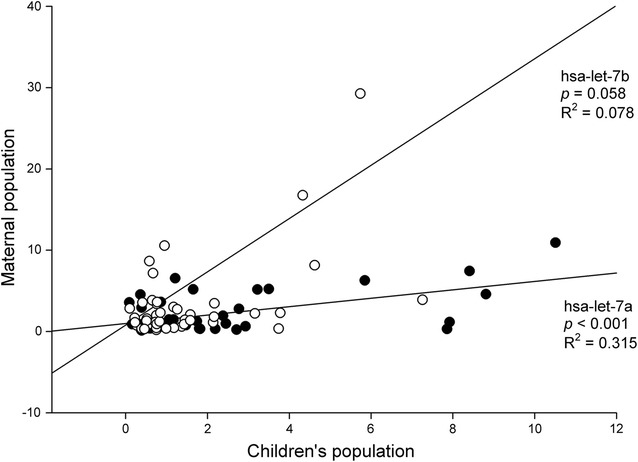
Association of hsa-let-7a/hsa-let-7b expression between children and maternal populations: A linear regression is established between expression levels of hsa-let-7a (solid circle)/hsa-let-7b (open circle) between children and maternal populations

As shown in Fig. 6, there was a significant correlation between hsa-let-7a levels in children and their mothers (R^2^ = 0.315, *p* < 0.001), while a marginal correlation was identified for hsa-let-7b levels (R^2^ = 0.078, *p* = 0.058). Hsa-miR-486 levels did not show a significant correlation between these two populations. In addition, we did not observe any significant associations of the miRNAs between the children and their fathers.

## Discussion

Through the screening of plasma miRNAs associated with cardiovascular development, we identified three miRNAs (hsa-let-7a, hsa-let-7b and hsa-miR-486) that were significantly upregulated in children with ASD. A further validation study showed that the overexpression of hsa-let-7a and hsa-let-7b was specifically present in children with ASD. ROC curve analyses suggested that both hsa-let-7a and hsa-let-7b had a significant diagnostic value for detecting ASD. Interestingly, a similar miRNA expression profile of hsa-let-7a and hsa-let-7b was discovered in the mothers of children with ASD and other types of CHD. Hsa-let-7a showed a high accuracy to distinguish mothers of offspring with ASD from mothers with healthy children. We conclude, therefore, that circulating miRNAs are highly feasible as biomarkers for clinical detection of CHD, and may be powerful and useful molecular tools for evaluating the risk of CHD in the next generation.

To our knowledge, there is paucity of data on the expression profile of circulating miRNAs in congenital heart malformations with ASD. Li et al. reported the up-regulation of miR-498 and down-regulation of miR-let- 7e-5p, miR-155-5p, miR-222-3p, miR379-5p, miR-433, and miR-487b, and miR-409-3p in plasma of pediatric patients with VSD [17]. Our newly identified three miRNAs are different from these molecules and we speculate that they are specific to the subtype of CHD. In this regard, we further examined the expression pattern of the three selected miRNAs in a larger sample size of children with ASD as well as children with VSD and VASD. Our data indicated that hsa-let-7a and hsa-let-7b were specifically upregulated in children with ASD, but not in children with VSD or AVSD, supporting our first hypothesis that specific miRNAs are associated with specific types of CHD. Such difference should be not so surprised as hsa-let-7a and hsa-let-7b were selected from miRNA array analysis using ASD samples. In contrast, a significant overexpression of hsa-miR-486 was observed in children with ASD, VSD or AVSD compared to the control children, indicating that hsa-miR-486 may be involved in a common molecular pathway that regulates the pathogenesis of different CHD types. This finding is in agreement with the notion that clinically distinct malformations can arise from single genetic defects, and unrelated cardiac structures are likely to share similar developmental pathways [23].

In analysing the diagnostic values of the three miRNAs using ROC curve analysis, we confirmed that hsa-miR-486 was a superior marker compared to hsa-let-7a and hsa-let-7b in diagnosing mixed phenotype CHD with an AUC value of 0.755 (sensitivity: 69%, specificity: 70%). Similarly, without considering the phenotypic heterogeneity of CHD, Zhu et al. identified four miRNAs (miR-19b, miR-22, miR-29c and miR-375) as biomarkers for the prenatal detection of fetal CHD, with AUC of 0.671–0.790 and sensitivity and specificity ranges 55.6–74.1% and 66.7–88.9% respectively [16]. Curti et al. reported AUC values of 0.503–0.804 using maternal plasma mRNA species for predicting fetal CHD with a detection rate of 66.7% and a false positive rate of 10% [24]. These reported diagnostic performances are comparable to our current finding for hsa-miR-486 as a common CHD marker. However, when we considered the specific types of CHD, we observed a significant improvement in discriminatory power, particularly by using hsa-let-7a and hsa-let-7b for detecting ASD patients. The ROC curve yielded an AUC value of 0.833 and a diagnostic sensitivity and specificity of 70 and 100% for hsa-let-7a, and AUC value of 0.900 and a diagnostic sensitivity and specificity of 91 and 90% for hsa-let-7b. These higher accuracy levels show that using specific biomarkers for CHD subtypes in clinical detection is necessary.

The parent-of-origin was proposed to be the etiological mechanism of CHD in children, including genetic/epigenetic and environmental influences [5]. The recent study performed in non-syndromic children CHD revealed significant enrichment of protein-truncating variants inherited from unaffected parents in CHD-associated genes [19]. One study even showed that the risk of CHD in children could be attributed to grandparents with cardiovascular disease [25]. In addition to the genetic influence, environmental factors in particular maternal lifestyle are highly related to risk of CHD. These include maternal smoking, alcohol, illicit drugs use, caffeine use, body mass index and psychological factors [26]. Concerning the underlying mechanisms of CHD, it is widely accepted that miRNAs play a critical role in regulating the pathogenesis of CHD. An increasing number of studies have shown that miRNAs also interactively regulate two other inheritable epigenetic components, namely DNA methylation and histone modification, to constitute an inter-regulatory system that assures an accurate transcriptional and translational expression of protein-coding genes [20]. In addition, the genetic differences in the overall efficiency of the miRNA biogenesis pathway were found between individuals, which were correlated to their parents [21]. As such, we reasonably speculate that miRNA signatures can be used as a genetic counseling tool to evaluate an individual’s risk of having CHD-affected offspring. Subsequently, we investigated the diagnostic values of these miRNAs for predicting CHD in offspring using parent–offspring trios. In these unaffected (healthy) parents, we observed similar changes in expression patterns, in hsa-let-7a and hsa-let-7b for mothers of probands, and in hsa-miR-486 for fathers of probands. ROC curve analyse showed a high accuracy of the hsa-let-7a level for differentiating mothers of children with ASD from mothers of healthy children (AUC: 0.917; sensitivity: 82%; specificity: 91%). A further correlation study demonstrated a positive association of hsa-let-7a and hsa-let-7b between children and their mothers. These data indicate that maternal effects are a major factor contributing to CHD development in the current cohort. The abnormal expression of hsa-miR-486 in the fathers of CHD children may be indicative of a paternal effect on the disease development. One important limitation of the present study is that we cannot ascertain the specific parental origin of the patients (maternal or paternal) and cause (genetic or environmental). This limits the data interpretation and hinders an accurate assessment of the predictive values. However, our data clearly suggest that miRNA signatures are promising biomarkers for determining the risk of CHD in offspring.

Interestingly, the let-7 family of circulating miRNAs is regarded as a mediator of intercellular communication [27]. In acute myocardial infarction (AMI) patients, let-7b is dramatically inhibited during the disease onset and of significant diagnostic value for AMI [28]. The circulating miR-486 regulation was found to depend on the tricuspid or bicuspid morphology of the aortic valve [29]. According to DIANA TOOLS [30], both let-7a and let-7b negatively regulate HAND1 mRNA with a miTG predictive score from 0.99 to 1.00. HAND1 governs the development of various tissues within the embryo, the extraembryonic mesoderm and trophectoderm. It is likely that the overexpression of let-7a and let-7b inhibits HAND1 and disrupts cardiac embryogenesis, which makes them promising diagnostic targets in detecting ASD. The downstream targets of miR-486 are predicted to be the *PTEN* and *FOXO1* genes. Both gene coding products are involved in the PI3-kinase/Akt signaling pathway, and consequently miR-486 is thought to regulate PI3-kinase/Akt signaling to cause muscle hypertrophic growth [31, 32]. Intriguingly, dysregulation of these miRNAs has not resulted in heart defects in the parental population, indicating that the functional effects of these miRNAs may depend on the critical development stages e.g. antenatal development. It is worthy of further investigation for understanding the fundamental mechanisms of CHD development.

## Conclusion

Overall, we identified a consistent expression pattern of circulating hsa-let-7a and hsa-let-7b in children with ASD and their mothers. These non-coding molecules in plasma are useful to diagnose children with ASD, and hsa-let-7a is a promising biomarker for evaluating the risk of ASD in offspring. Our data support the potential of using circulating miRNAs as diagnostic and genetic screening markers for CHD. However, the sample size is small in the present study, particularly after stratifying by CHD phenotype. Large-scale studies are needed to replicate our results and further validate the candidate biomarker specificity by including other cardiovascular diseases. Additionally, it is of great interest to test the suitability of these markers for prenatal screening or prognosis evaluation of CHD. Finally, in light of our findings, we strongly recommend that specific miRNAs should be explored in each type of CHD and the multiple specific miRNAs should be ultimately combined into one biomarker panel to aid in the clinical diagnosis, screening and prediction of CHD.

## Abbreviations

CHD: congenital heart defects
miRNAs: microRNAs
ASD: atrial septal defects
VSD: ventricular septal defects
AVSD: atrioventricular septal defects
qPCR: quantitative real-time PCR
ROC: receiver-operating characteristic
AUC: area under the ROC curve
AMI: acute myocardial infarction
